# A new automated and putatively versatile synthesis of the PSMA-ligand derivative [^18^F]DCFPyL using the FASTlab^TM^ synthesizer

**DOI:** 10.1186/s41181-022-00157-0

**Published:** 2022-05-04

**Authors:** Raphaël Hoareau, Tore Bach-Gansmo, Paul Cumming, Dag Erlend Olberg

**Affiliations:** 1 Universitetssjukhuset Örebro, Örebro, Sweden; 2 Oslo University Hospital, Oslo, Norway; 3 School of Psychology and Counselling, Queensland University of Technology, Brisbane, Australia; 4 Institute of Nuclear Medicine, Inelspital, University of Bern, Bern, Switzerland; 5 Norsk Medisinsk Syklotronsenter AS, Postboks 4950, 0424 Nydalen, Oslo, Norway; 6 School of Pharmacy, University of Oslo, Oslo, Norway

**Keywords:** Rapid, PSMA, Biomolecules, On-cartridge radiolabeling, Prosthetic group, Automation, DCFPyL

## Abstract

**Background:**

Noninvasive molecular imaging using peptides and biomolecules labelled with positron emitters has become important for detection of cancer and other diseases with PET (positron emission tomography). The positron emitting radionuclide fluorine-18 is widely available in high yield from cyclotrons and has favorable decay (t_1/2_ 109.7 min) and imaging properties. ^18^F-Labelling of biomolecules and peptides for use as radiotracers is customarily achieved in a two-step approach, which can be challenging to automate. 6-[^18^F]Fluoronicotinic acid 2,3,5,6-tetrafluorophenyl ester ([^18^F]F-Py-TFP) is a versatile ^18^F-prosthetic group for this purpose, which can be rapidly be produced in an one-step approach on solid support. This work details an automated procedure on the cassette-based GE FASTlab™ platform for the labeling of a peptidomimetic, exemplified by the case of using the Glu-CO-Lys motif to produce [^18^F]DCFPyL, a ligand targeting the prostate specific membrane antigen (PSMA).

**Results:**

From fluorine-18 delivery a fully automated two-step radiosynthesis of [^18^F]DCFPyL was completed in 56 min with an overall end of synthesis yield as high as 37% using solid phase extraction (SPE) purification on the GE FASTlab™ platform.

**Conclusions:**

Putatively, this radiolabeling methodology is inherently amenable to automation with a diverse set of synthesis modules, and it should generalize for production of a broad spectrum of biomolecule-based radiotracers for use in PET imaging.

## Background

Cancer is the leading cause of premature deaths in the western world, a circumstance that has spurred the development of new radiopharmaceuticals for cancer diagnosis by positron emission tomography (PET) and for radiotherapy using α- and β-emitting radionuclides (Bouchelouche et al. 2010; Holland et al. 2012; Zhang et al. 2017). For use in clinical PET imaging, the radiohalogen fluorine-18 (^18^F) is the preferred radioisotope due to its physical half-life (109.7 min), favorable decay properties, and its ready availability in multi-GBq quantities from medical cyclotrons. A considerable proportion of emerging radiopharmaceuticals are based on small to medium sized peptides, fusion proteins, and endogenous proteins. However, these products are not always obtainable through a direct radiolabeling approach due to their instability at high temperature or the otherwise harsh reaction conditions required to introduce fluorine-18 into their structure. Although labeling of such molecules with fluorine-18 using a chelator conjugated to the targeting moiety with [^18^F]AlF (aluminum [^18^F]fluoride) is feasible, this approach can release free [^18^F]fluoride in vivo (which invariably results in intense bone labelling) and otherwise alter the biodistribution properties of the radiopharmaceutical in an unpredictable fashion (Lütje et al. 2019).

**Fig. 1:**
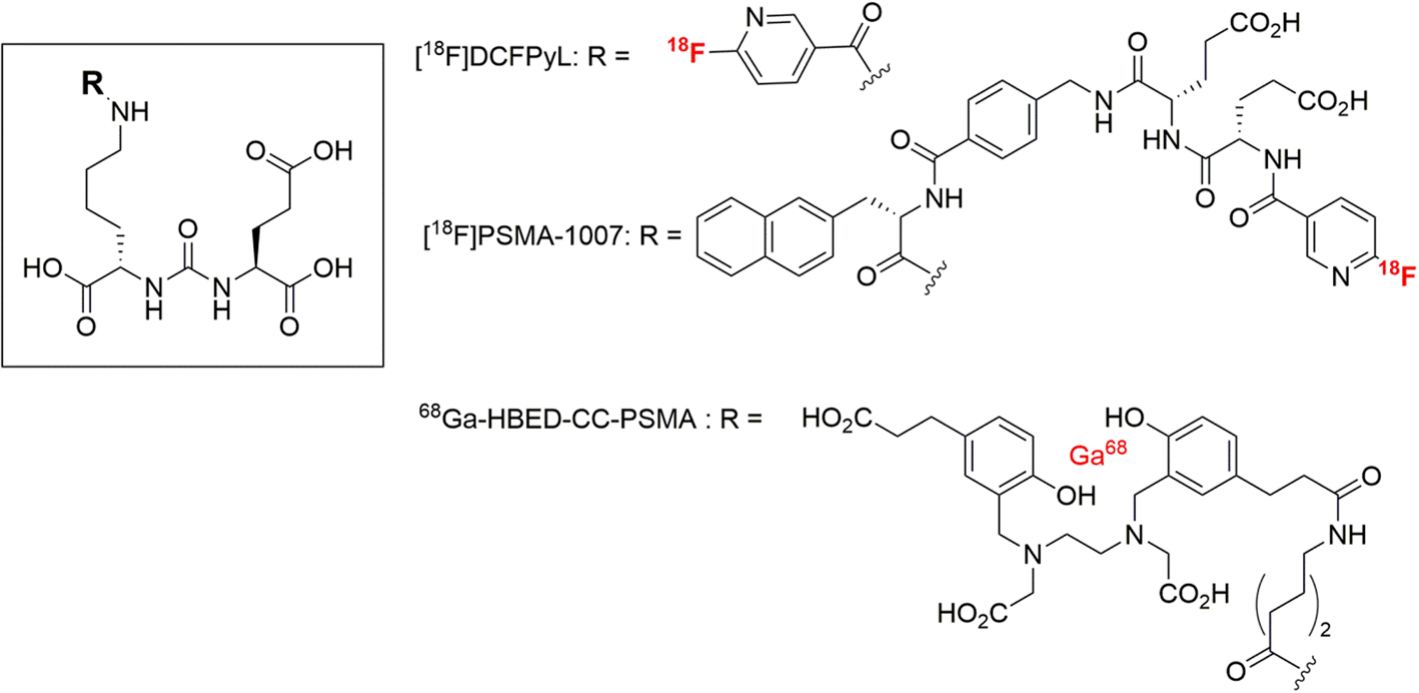
Three Glu-CO-Lys-based PET radiopharmaceuticals presently in clinical use for PSMA imaging

A robust and high yield radiosynthesis amenable to automation is a prerequisite for adopting a PET radiopharmaceutical for widespread clinical or research use (Li et al. 2013). Production of PET radiopharmaceuticals with a multi-step synthetic process can be a challenging task using commercially produced cassette-based modules, mainly due to their lack of modifiability, and the frequent requirement for intermediate or final HPLC purification. ^18^F-labelled tracers for prostate specific membrane antigen (PSMA) [^18^F]DCFPyL and [^18^F]-PSMA-1007 are examples of PET radiopharmaceuticals that formerly required a two-step radiosynthesis from the prosthetic group 6‐[^18^F]fluoronicotinic acid‐2,3,5,6‐tetrafluorophenyl ester ([^18^F]F‐Py‐TFP) (Chen et al. 2011; Giesel et al. 2017, Cardinale et al. 2017). In the case of [^18^F]DCFPyL, the first reported radiosynthesis from [^18^F]F‐Py‐TFP and the PSMA-avid substrate Glu-CO-Lys protected with *t*-butyl has subsequently been optimized (Chen et al. 2011). Currently, a direct precursor labeling with the trimethylammonium nicotinic acid moiety conjugated to the ε-nitrogen of the lysine residue of Glu-CO-Lys is the favored method for automated multi-dose production of [^18^F]DCFPyL (Dornan et al. 2018), which has greatly facilitated the use of PSMA PET in clinical routine (Fig. 1). However, this introduction of fluoride-18 in organic solvents at a temperature approaching 100 °C, is unlikely to provide high labeling efficiencies, or to generalize for larger molecular weight radiopharmaceuticals such as polypeptides and proteins with their bountiful functional groups.

Fairly recently, a simplistic and rapid preparation of [^18^F]F‐Py‐TFP has been reported, wherein the prosthetic group is obtained directly by passing its precursor in a solution of acetonitrile/*tert*-butanol through a polymeric anion‐exchange cartridge preloaded with [^18^F]fluoride (Basuli et al. 2016; Olberg and Svadeberg 2017). This procedure yields the bifunctional labeling agent in good yield, with an overall synthesis time of less than ten minutes (EOB), simultaneously avoiding the use of a reaction vessel and a phase transfer catalyst. The on-column radiosynthesis of [^18^F]F‐Py‐TFP has a very small laboratory footprint and lends itself well for automation and offer radiolabeling of a wide variety of sensitive biomolecules (proteins eg.) (Roy et al. 2019; Lesniak et al. 2019). Expanding on this concept, we report a fully automated, two-step radiosynthesis of [^18^F]DCFPyL from [^18^F]F‐Py-TFP and the Glu-CO-Lys pseudo-peptide precursor, which can serve as a blueprint manufacturing process for the preparation of other ^18^F-labelled radiopharmaceuticals. The approach uses [^18^F]F‐Py‐TFP produced on column adopted into the commercially available, cassette-based synthesis module FASTlab™. The automated process implements a hydrophilic interaction liquid chromatography (HILIC) SPE cartridge purification methodology, making final preparative HPLC purification redundant.

## Methods

### General

Chemicals were obtained either from Sigma-Aldrich (Norway) or from VWR (Norway), which were used without further purification. HPLC grade water was obtained from the Direct-Q® (EMD Millipore) water purification system. Reference standard 6-fluoronicotinic acid 2,3,5,6-tetrafluorophenyl ester (**1**) and its trimethylammonium precursor (**2**) were synthesized according to literature methods (Olberg et al. 2010). DCFPyL (2-(3-{1-carboxy-5-[(6-fluoro-pyridine-3-carbonyl)-amino]-pentyl}-ureido)-pentanedioic acid) (**3**) reference standard was purchased from ABX GmbH (Radeberg, Germany). Glu-CO-Lys (2-[3-(5-Amino-1-carboxy-pentyl)-ureido]-pentanedioic acid trifluoroacetate salt) (**4**) was purchased from ABX GmbH or Scintomics GmbH (Fürstenfeldbruck, Germany). Sep-Pak™ cartridges were purchased from Waters (Norway). HILIC cartridges containing 350 mg of Chromabond® HILIC sorbent (Macherey–Nagel GmbH, Düren) and anion-exchange columns containing 45 mg of Chromabond® PS-HCO_3_, (Macherey–Nagel GmbH) were obtained from Teknolab AS (Ski, Norway). Analytical HPLC was performed on an Agilent 1100 series HPLC system with UV detection connected in series with a NaI γ-radiodetector (Institute for Energy Technology (IFE), Kjeller, Norway). Samples were analyzed using methods **1** or **2a** and **b** as described below. Chromatograms were processed with Agilent OpenLAB CDS EZChrom version A.04.04. Reversed phased-analytical HPLC was performed on a Phenomenex kinetex EVO C18 column (50 × 2.1 mm, 2.6 µm particles), at a flow rate of 0.5 mL/min, with a composition gradient from 2–95% solvent B (method **1**) over ten min (solvent A: water/0.05% TFA, solvent B: acetonitrile/0.05% TFA). UV detection was at 214, 254 and 264 nm. Hydrophilic interaction liquid chromatography (HILIC) was performed on a SeQuant ZIC-cHILIC (100 × 4.6 mm, 3 µm particles) at a flow rate of 2.0 mL/min, with composition gradient from 2–98% solvent B (method **2a**) or isocratically with 68% solvent B (method **2b**) over ten min (Solvent A: 97% acetonitrile/3% 5 mM NH_4_OAc, solvent B: 5 mM NH_4_OAc). UV detection was at 210, 220, 254 and 264 nm. Thin layer chromatography (TLC) was run on gel 60F_254_ plates (Merck) using acetonitrile as eluent. A Raytest miniGita (Raytest, Germany) equipped with a β-detector was used to record the radio-TLC scan. Gas chromatography (GC) was performed with an Agilent 6890 N GC (Matriks, Norway) equipped with a flame ionization detector (FID), a column oven, and an auto-sampler with a direct injection system. The GC column was a fused silica capillary column with USP stationary phase G43 (6% cyanopropylphenyl—94% dimethyl polysiloxane) measuring 0.32 mm i.d. × 30 m. Automated radiochemistry was performed on the GE FASTlab synthesizer with the custom-made single-use FASTlab development kit (GE Healthcare) placed inside a lead-shielded hot cell. Fluorine-18 was produced in a GE PETtrace 6 cyclotron (General Electric Healthcare) with a GE ^18^F^−^ Nb 27 self-shielded target either by irradiation (40 µAh × two min) of [^18^O]H_2_O (Taiyo Nippon Sanso, Japan), or from a target rinse after a routine 100 GBq production. No more than 150 MBq of radioactivity were handled outside the hot cell. Radioactivity was measured with a Capintec dose calibrator (New Jersey, USA). The identities of the radiolabeled products were confirmed by co-injection to HPLC along with authentic nonradioactive standards.

### General radiolabeling procedure (manual)

Aqueous [^18^F]fluoride was trapped on a Chromabond PS-HCO_3_ anion cartridge followed by rinsing with hot acetonitrile (2 mL) and passage of air (2 × 5 mL) to dryness. A solution of precursor **2** (10 mg/mL, 22 mM), in the presence of TEA in tert-butanol (*t*-BuOH)/acetonitrile (50%, v/v, 1 mL) was used to initiate radiolabeling and to elute the radioactivity into a receiving vial (10 s); the eluent was passed through the column back and forth into the syringe barrel for 20 s. After the final elution into the vial, the reaction was quenched by rinsing the column with water (0.5 mL). We measured the radioactivity concentration in the quenched reaction mixture before analysis by HPLC and TLC with on-line gamma detection.

### General automated radiolabeling procedure

**Fig. 2:**
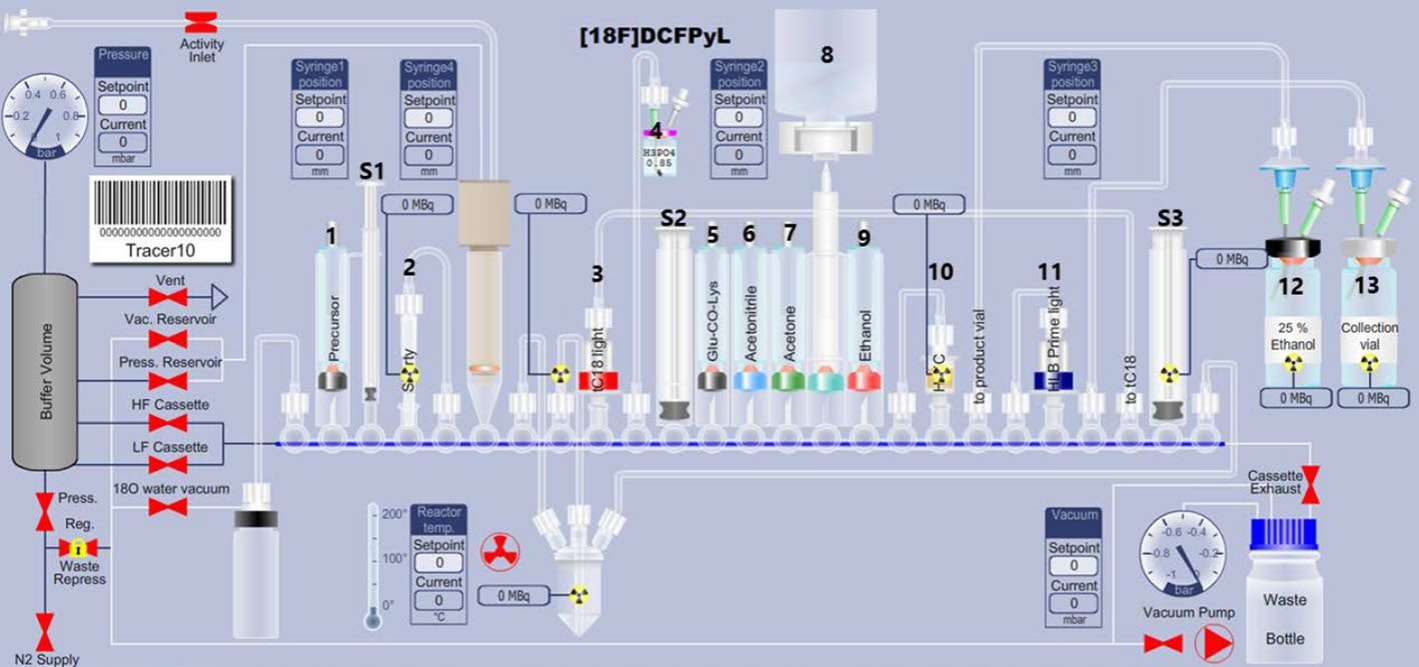
Layout of the [^18^F]DCFPyL FASTlab cassette. **1.** Precursor vial **2.** Chromabond PS-HCO_3_ cartridge **3.** Sep-Pak tC18 plus light cartridge **4.** 0.85% H_3_PO_4_ external vial **5.** Glu-CO-Lys vial **6.** Acetonitrile **7.** Acetone **8.** Water bag **9.** Abs. ethanol **10.** HILIC SPE cartridge **11**. Oasis PRiME HLB plus light cartridge **12.** 25% ethanolic solution external vial **13.** Product collection vial. **S1**, **S2** and **S3**, syringes 1, 2 and 3

Cassettes were assembled according to the layout depicted in Fig. 2. External vials were vented with a syringe filter and connected to the cassette manifold with a silicone tube of specific length, terminating in a needle as follows:

- 30 mL vial of H_3_PO_4_ 0.85%: 42-cm long tube, 0.80 i.d. × 80 mm needle (vial 4)
- Collection vial (10 mL) 13-cm long tube, 0.80 i.d. × 80 mm needle (vial 13)
- 25% ethanol vial: 13-cm long tube, 2.10 mm i.d. × 80 mm needle (vial 12)

Vials were prepared as follows:

- Precursor vial: [^18^F]F-Py-TFP precursor **2** (15 mg, 31.4 µmol) in *t*-BuOH/acetonitrile (1.4 mL) and triethylamine (TEA) (0.4 eq.) (vial 1)
- Glu-CO-lys vial: Glu-CO-lys (13.2 mg, 29.1 µmol), TEA-HCO_3_ (4.3 eq.) in 0.63 mL DMSO (vial 5)
- Acetonitrile vial: 4.0 mL (vial 6)
- Acetone and absolute ethanol vials: 4.6 mL each (vial 7 and vial 9)

## Results

From the get-go, reaction conditions were investigated manually, aiming to determine suitable and robust conditions for on-cartridge synthesis of [^18^F]F-Py-TFP for translation to the automated synthesis. In brief, fluorine-18 was trapped on the Chromabond PS-HCO_3_ and then the column was dried with anhydrous acetonitrile. Fluorination occurred on-cartridge when the mixture of precursor **2** and base in acetonitrile/t-BuOH was cycled through the cartridge. The radiochemical yield is dependent of precursor concentration and the presence of an non-nucleophilic organic base helps to increase yield and reduce variantion between synthesis runs.

RCYs improved when the Chromabond column was dried using acetonitrile heated to 80 °C in the reactor vessel. The best RCY (78 ± 9%) was obtained using 11 mM of precursor and 0.2 equivalents of triethylamin (TEA) as the base. Translating these conditions (including SPE purification) into the FASTlab, [^18^F]F-Py-TFP was obtained with RCY of 63 ± 4% with TEA as the base. In our hands, yields were 15% lower using the automated FASTlab synthesis compared to manual synthesis and might be due to the longer fluid paths in the automated synthesis. For the automated FASTlab [^18^F]DCFPyL synthesis, 22 mM of precursor **2** and 8 mM of TEA in 1.0 mL acetonitrile/t-BuOH (50/50 v/v) was used routinly throug this work.

The automated radiosynthesis entailed three sequential processes, i.e. a ten min pre-labelling step, a 25 min on-cartridge radiolabeling step for [^18^F]F-Py-TFP (including purification), and a 31 min conjugation and purification step affording the final product [^18^F]DCFPyL (56 min in total). Due to the inherent dead volumes, when implementing the radiochemistry to the FASTlab synthesizer, the reagent vials were filled with slightly larger volumes to retain the same stoichiometric amounts of reagents.

### Pre-labelling

The vials, cartridges and reactor were conditioned for ten minutes before delivery of radioactivity. Vials were pressurized with 1 bar nitrogen to ensure optimal syringe filling. Ethanol and water were successively used to condition the tC18 Sep-Pak light cartridge. Acetonitrile (1.6 mL) was transferred into the FASTlab reactor and heated; the system was deemed ready to receive radioactivity when the reactor temperature reached 80 °C.

### Radiolabeling and purification of [^18^F]F-Py-TFP

**Fig. 3:**
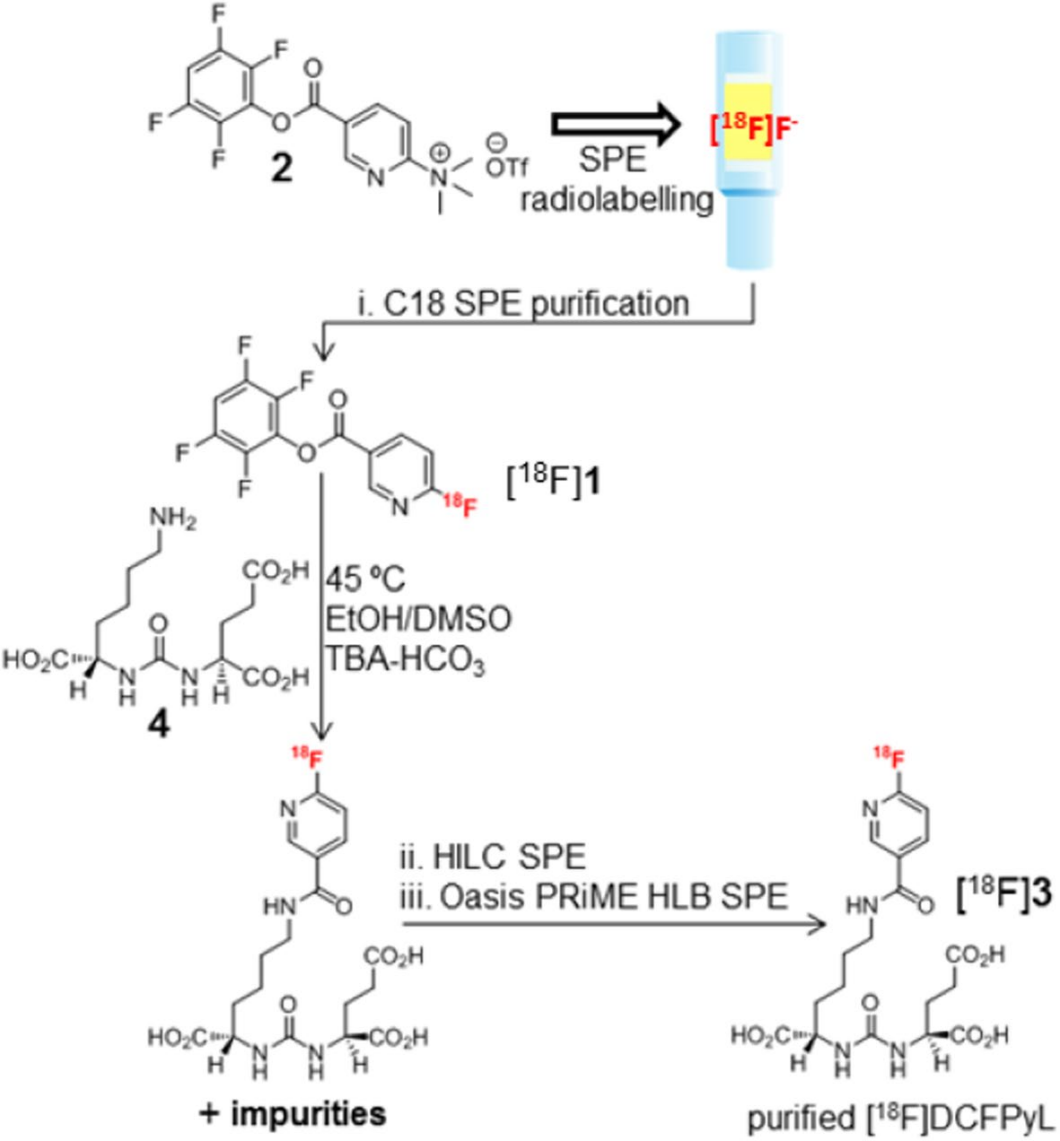
Overview of the radiosynthesis process on the FASTlab

The next step provided purified [^18^F]F-Py-TFP bound to the tC18 Sep-Pak light cartridge in 25 min. First, fluorine-18 was trapped in the Chromabond PS-HCO_3_ cartridge, followed by drying with two passages of hot acetonitrile drawn from the heated reactor. The acetonitrile was successively sent to syringes 2 and 3 to remove any water remaining from the pre-labeling step. Using syringe 1, a 1 mL volume of the precursor solution was pushed through the Chromabond PS-HCO_3_ cartridge over a period of 45 s, with a series of slow back and forth strokes. The radioactive mixture was then transferred into syringe 2 and quenched by rinsing of the Chromabond PS-HCO_3_ with water. Syringe 2 was further loaded with two additional aqueous rinses from the Chromabond PS-HCO_3_ cartridge. Radiochemically pure [^18^F]F-Py-TFP was obtained by diluting the organic solvent content with water to 20% and extracting the [^18^F]F-Py-TFP onto the tC18 Sep-Pak light cartridge (flow 2 mL/min), which was followed by a 6 mL water rinse of the tC18 via syringe 2. Partially dry SPE-bound [^18^F]F-Py-TFP was obtained by purging the SPE cartridge with a stream of nitrogen gas (N_2_) at high flow rate. Figure 3 and Table 1 present an overview of the cassette synthesis and the impurities removed by each of the SPE cartridges used in the FASTlab cassette.

**Table 1:** Overview of impurities removed at each SPE-purification step

Entry^a^	Purification cartridge	Impurities removed by cartridge
i	Sep-Pak light cartridge	[^18^F]Fluoride, unreacted F-Py-TFP precursor, and hydrolyzed F-Py-TFP precursor
ii	HILIC cartridge	Unreacted [^18^F]F-Py-TFP,
iii	Oasis HLB PRiME cartridge	

^a^ Based on synthetic scheme numbering (i, ii, iii) in Fig. 3

### Reaction of [^18^F]F-Py-TFP to Glu-CO-Lys and purification of [^18^F]DCFPyL

From cartridge-bound [^18^F]F-Py-TFP, [^18^F]DCFPyL was synthesized and purified in 31 min.

The reactor was charged with Glu-CO-Lys/TBA-HCO_3_ in DMSO by applying vacuum, and the [^18^F]F-Py-TFP was then quantitatively eluted from the tC18 Sep-Pak light cartridge into the reactor using 0.7 mL absolute ethanol. Aminolysis of [^18^F]F-Py-TFP with Glu-CO-Lys was performed at 45 °C in the presence of TBA-HCO_3_ as base for 10 min, providing crude [^18^F]DCFPyL. In parallel, the cassette manifold was prepared for the final purification step. The HILIC and Oasis PRiME HLB plus light cartridges were both first conditioned with water from syringe 3. Syringe 2 was first rinsed with ethanol; (1 mL) and then acetone (1.2 mL), which was used to condition the HILIC. After conjugation, the reaction mixture was diluted with acetone (2.4 mL) and transferred via syringe 2 to the HILIC cartridge, immobilizing the [^18^F]DCFPyL product while more apolar impurities were unretained. Residual radioactivity was transferred from the reactor to the HILIC cartridge using a second portion of acetone (1.0 mL). The remaining acetone in vial 7 (approx. 1 mL) was used to rinse the HILIC cartridge followed by a N_2_-gas purge. Syringe 2 was rinsed with 0.85% phosphoric acid, and a 4.2 mL volume of this same acid solution was used to elute the [^18^F]DCFPyL fraction off the HILIC column to syringe 3, which was further diluted with 2.7 mL of 0.85% phosphoric acid.

Considering that both [^18^F]DCFPyL and unreacted precursor Glu-CO-Lys were retained by the HILIC SPE and both was eluted with weak phosphoric acid, an additional purification step to remove unreacted Glu-CO-Lys, which is a pseudo-carrier possessing PSMA receptor avidity, was required. After investigating different reverse solid-phase materials, including C18 and other polymeric phases, we identified the Oasis PRiME HLB plus light cartridge as being suitable, by retaining [^18^F]DCFPyL but not the more polar Glu-CO-Lys. Consequently, [^18^F]DCFPyL could be trapped selectively on the Oasis PRiME HLB light cartridge using syringe 2 as a waste receiver. The cartridge was washed with an additional 4.8 mL of phosphoric acid to elute any residual Glu-CO-Lys. Finally, syringe 3 was washed three times with water and [^18^F]DCFPyL was eluted off the Oasis PRiME cartridge into the product vial with 2.1 mL 25% ethanolic solution.

### Quality control of the drug product

**Fig. 4:**
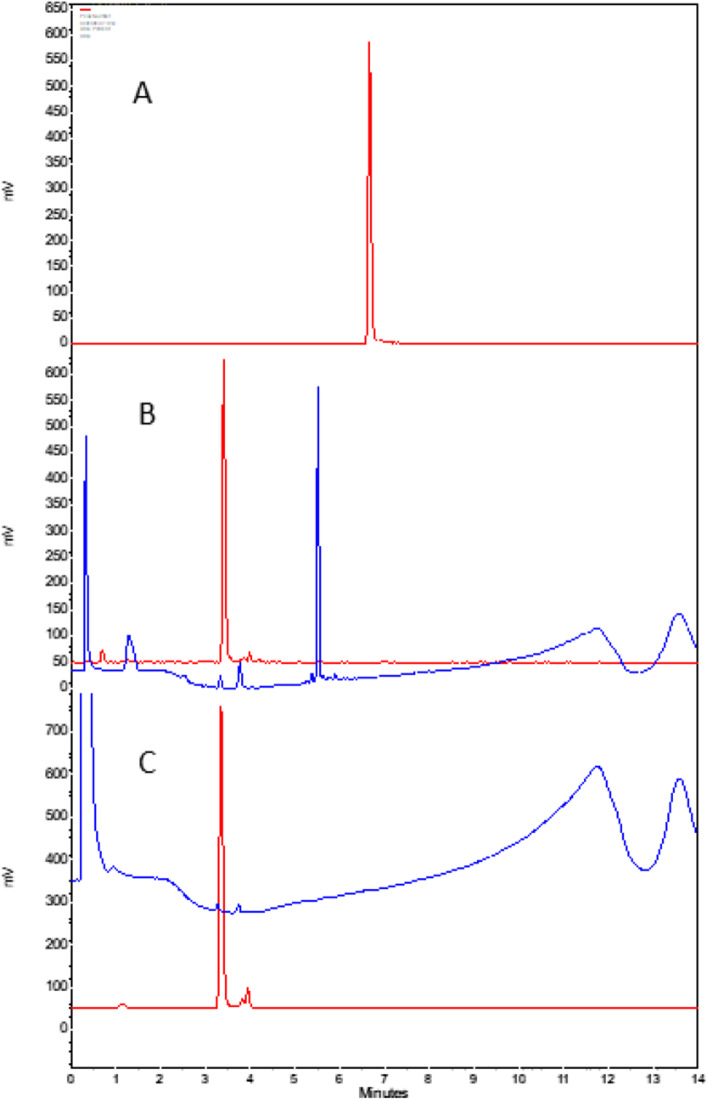
HPLC chromatograms at separate stages of the radiosynthesis **A** HPLC analysis of [^18^F]F-Py-TFP automated on the FASTlab synthesizer. Solid red line; radiodetector (Method 1). **B** Reaction mixture after conjugation of [^18^F]F-Py-TFP with Glu-CO-Lys (**4**) prior to SPE cartridge purification from the FASTlab synthesizer. Solid red line; radiodetector. Solid blue line; UV detection at 254 nm. **C** HPLC analysis of SPE cartridge purified [^18^F]DCFPyL from a fully automated radiosynthesis using the FASTlab synthesizer. Solid red line; radiodetector. Solid blue line; UV detection at 254 nm

HPLC method **1** (reversed phase) was used in conjunction with on-line radioactivity detection for routine assessment of radiochemical and chemical purity. Chemical and radiochemical purity (RCP) of the undiluted product was also monitored using method **2a**. The HILIC HPLC method **2b** was used for quantifying in the purified product any residual Glu-CO-Lys, which was too hydrophilic to be resolved by reversed phased HPLC. Representative chromatograms from individual steps of the automated synthesis are depicted in Fig. 4. The results for four automated runs, using starting activities ranging from 1 to 95 GBq along with low activity automated runs (≤ 400 MBq), are shown in Table 2. The radiochemical yield was close to 40% irrespective of the starting activity using starting activities from 1 GBq and upwards. Furthermore, the radiochemical purity was never lower than 93% (range 93–98%) at end of synthesis (Table 3), and declined in the first hours after high radioactivity productions, in agreement with already published work showing radiolysis of multi-GBq productions of [^18^F]DCFPyL (Ravert et al. 2016). Dilution and final formulation of the drug product to reduce RAC and inclusion of a scavenger such as sodium ascorbate, is expected to stabilize RCP further.

**Table 2:** FASTlab process yield of the automated synthesis of [^18^F]DCFPyL

Staring activity	250–400 MBq*	< 1 GBq	10 GBq	50 GBq	95 GBq
Isolated yield (EOS)	89.4–153.6**	0.24	2.4	14.6	24.4
RCY (%)***	56.3 ± 2.8	39	42	43	38

*n = 3

**Range in MBq

***Decay corrected yield (56 min synthesis time)

**Table 3:** Main quality control parameters at different starting activities

Staring activity EOB	< 1 GBq	10 GBq	50 GBq	95 GBq
Radio chemical purity (RCP) (%)	93	98	96	94
Total DCFPyL µmol (µg)	0.1 (41)	1.1 (47)	0.23 (103)	0.28 (124)
Total mass (µg)*	56	61	128	170
Molar activity (GBq/µmol)	18	48	92	123

*Total mass in 2.1 mL volume

## Discussion

In the final radiopharmaceutical product, carrier DCFPyL is the main constituent of the PSMA-avid impurities; its concentration increases with higher starting radioactivities. The total chemical impurities were well below 200 µg per batch, such that dilution and formulation to a final 20 mL volume drug product would give concentration of less than 10 µg/mL. This is below the chemical purity specifications adopted for PSMA-1007 (sum of all impurities bellow 0.5 mg/V_max_ and 0.1 mg/V_max_ for carrier PSMA-1007) (Cardinale et al. 2017). Although the chemical impurities in this work is higher than in [^18^F]DCFPyL productions where final purification was performed with HPLC (20 nmol/mL [sum of all impurities] vs. a maximum of 0.88 nmol/mL [sum all unknown impurities for HPLC-purified [^18^F]DCFPyL]) (Windhorst 2019). The overall molar activity was moderately high, increasing sevenfold as starting radioactivity increased from 1 to 95 GBq. Nevertheless, the entire range of obtained molar activity is suitable for PET imaging of highly expressed receptor targets such as PSMA (7 GBq/µmol) (Pillarsetty et al. 2016). Residual solvents in the final drug product (acetone, TEA, acetonitrile, DMSO and *t*-BuOH) where quantified using GC-FID and were found to be well below the Class II (acetonitrile) and Class III residual solvent limits specified in the European Pharmacopeia.

## Conclusion

We developed a fully automated two-step radiosynthesis of the PSMA ligand [^18^F]DCFPyL employing the prosthetic group [^18^F]-Py-TFP produced on-cartridge followed by conjugation to the PSMA-binding moiety Glu-CO-Lys. The radiosynhesis omits a preparative HPLC purification step. Our adoption of this reaction in the FASTlab™ synthesiser affords convenient access to the PSMA tracer [^18^F]DCFPyL in radiochemical yields ranging from 25 to 37% (uncorrected). The process, using GMP compliant single-use cassettes, afforded [^18^F]DCFPyL in adequate radiochemical purity (93–98%) and molar activity (70 ± 47 GBq/µmol), with radiochemical yields sufficient to support multiple clinical PET investigations from a single production. Residual solvents were well below the concentration limits adopted in the current version of the European Pharmacopeia. Furthermore, the fully automated proof-of-concept two-step process bodes well for a more general application to other radiosynthesis modules and for conjugation with large biomolecules such as oligopeptides and proteins.

## Data Availability

The datasets used and/or analyzed during the current study are available from the corresponding author on reasonable request.

## Abbreviations

PSMA: Prostate specific membrane antigen
[^18^F]F‐Py‐TFP: [^18^F]fluoronicotinic acid‐2,3,5,6‐tetrafluorophenyl ester
[^18^F]AlF: Aluminum [^18^F]fluoride
RCP: Radiochemical purity
EOS: End of synthesis
*t*-BuOH: Tert-butanol
SPE: Solid phase extraction
TEA: Triethylamine
TFA: Trifluoroacetic acid
GC: Gas chromatography
FID: Flame ionization detector
TBA-HCO_3_: Tetrabutylammonium carbonate
TLC: Thin layer chromatography
